# Supplementary material for: Organotypic Culture of Testicular Tissue from Infant Boys with Cryptorchidism
Source: Int J Mol Sci. 2022 Jul 19;23(14):7975. doi: 10.3390/ijms23147975 (PMC9316019; doi:10.3390/ijms23147975)
Supplement: Supplementary file 1 [file ijms-23-07975-s001.zip › Supplementary materials.pdf]

International Journal of
Molecular Sciences

MDPI

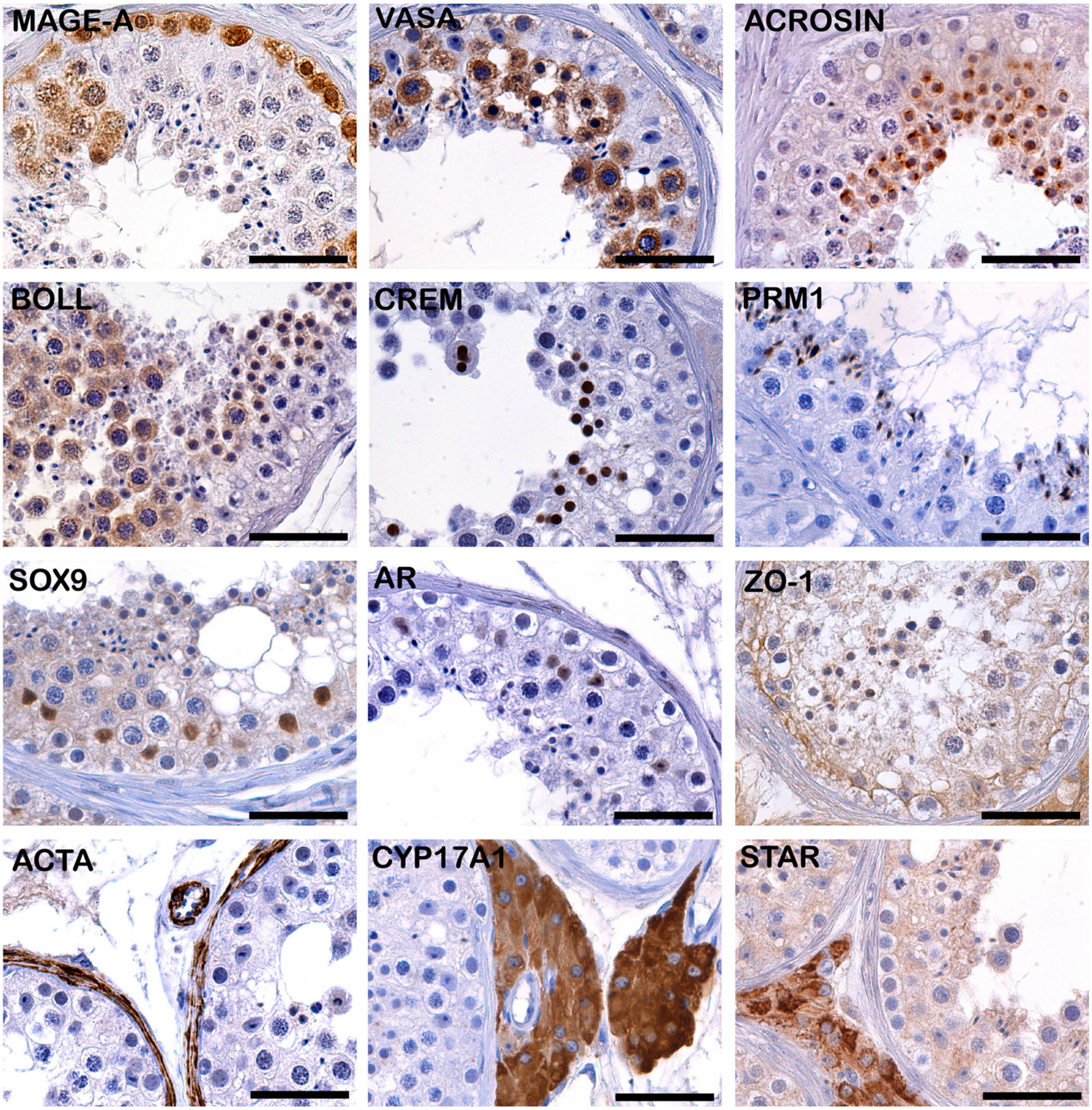

Figure S1 Expression of germ cell and somatic cell markers within normal adult testicular tissues which were fixed in Bouin. Germ cell markers: positive expression of MAGE-A at spermatogonia and some spermatocytes; positive expression of VASA at spermatogonia, spermatocytes, and round spermatids; positive expression of acrosin at round spermatids; positive expression of BOLL at spermatocytes and round spermatids; positive expression of CREM at round spermatids; positive expression of PRM1 at elongated spermatids. Sertoli cell markers: SOX9 and AR. Blood-testis barrier marker ZO-1. PTMCs maker ACTA. Leydig cell markers CYP17A1 and STAR. Brown color indicated positive expression of different markers. Scale bar=50μm.

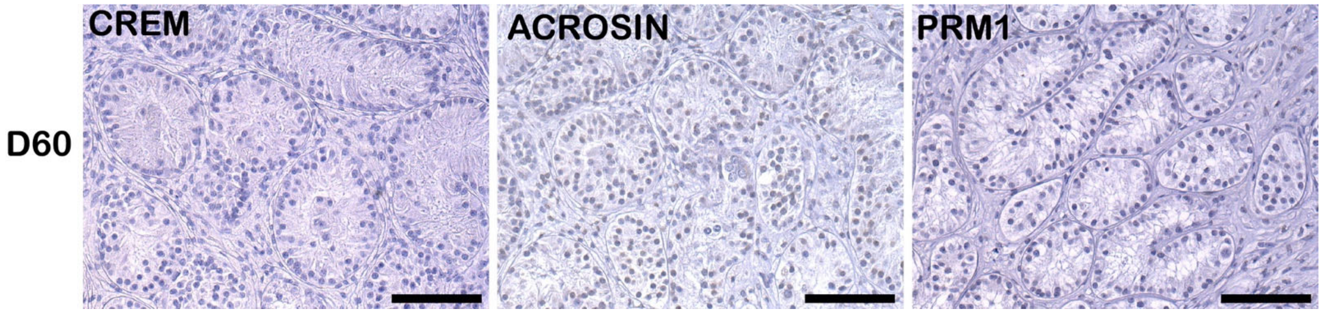

Figure S2 Negative expression of CREM, ACROSIN, and PRM1 within testicular tissues during the 60-day culture. Scale bar=100μm.

Table S1 Primary antibodies and target cells

Antibody	Target cells	Concentration	Catalog number	Manufacturer
MAGEA	spermatogonia	1:200	sc-20034	Santa Cruz
GAGE	spermatogonia	1:150	G13520	Transduction
VASA	spermatogonia	1:100	AF2030	R&D SYSTEMS
SOX9	Sertoli cells	1:100	AB5535	Merck Millipore
AMH	immature Sertoli cell	1:100	AF2748	R&D systems
AR	mature Sertoli cell	1:100	ab108341	abcam
ZO-1	tight junction	1:200	617300	Thermo-Fisher
ACTA	peritubular myoid cell	1:150	ab5694	abcam
Ki67	proliferating cell	1:50	M7240	DAKO
BOLL	spermatocyte and spermatid	1:100	HPA048813	Sigma-Aldrich
ACROSIN	round spermatid	1:100	HPA048687	ATLAS SYSTEMS
CREM	round spermatid	1:500	HPA001818	Sigma-Aldrich
PRM1	elongated spermatid	1:200	HPA055150	Sigma-Aldrich
CYP17A1	Leydig cell	1:200	sc-46084	Santa Cruz
STAR	Leydig cell	1:200	sc-166821	Santa Cruz
